# Dot Immunobinding Assay for the Rapid Serodetection of *Scedosporium*/*Lomentospora* in Cystic Fibrosis Patients

**DOI:** 10.3390/jof9020158

**Published:** 2023-01-24

**Authors:** Leire Martin-Souto, Aitziber Antoran, Maialen Areitio, Leire Aparicio-Fernandez, María Teresa Martín-Gómez, Roberto Fernandez, Egoitz Astigarraga, Gabriel Barreda-Gómez, Carsten Schwarz, Volker Rickerts, Fernando L. Hernando, Aitor Rementeria, Idoia Buldain, Andoni Ramirez-Garcia

**Affiliations:** 1 Department of Immunology, Microbiology and Parasitology, Faculty of Science and Technology, University of the Basque Country (UPV/EHU), 48940 Leioa, Spain; 2 Microbiology Department, Vall d’Hebron University Hospital, 08035 Barcelona, Spain; 3 Department of Research and Development, IMG Pharma Biotech S.L., 48160 Derio, Spain; 4 Division of Cystic Fibrosis, CF Center Westbrandenburg, Campus Potsdam, Klinikum Westbrandenburg, 14467 Potsdam, Germany; 5 Faculty of Medicine, HMU-Health and Medical University Potsdam, 14471 Potsdam, Germany; 6 Charité-Universitaetsmedizin Berlin, 10117 Berlin, Germany; 7 Division for Mycotic and Parasitic Agents and Mycobacteria, Robert Koch Institute Berlin, 13353 Berlin, Germany

**Keywords:** *Scedosporium*, *Lomentospora*, cystic fibrosis, immunoassay, serological detection, antibody detection

## Abstract

The detection of *Scedosporium*/*Lomentospora* is still based on non-standardized low-sensitivity culture procedures. This fact is particularly worrying in patients with cystic fibrosis (CF), where these fungi are the second most common filamentous fungi isolated, because a poor and delayed diagnosis can worsen the prognosis of the disease. To contribute to the discovery of new diagnostic strategies, a rapid serological dot immunobinding assay (DIA) that allows the detection of serum IgG against *Scedosporium*/*Lomentospora* in less than 15 min was developed. A crude protein extract from the conidia and hyphae of *Scedosporium boydii* was employed as a fungal antigen. The DIA was evaluated using 303 CF serum samples (162 patients) grouped according to the detection of *Scedosporium*/*Lomentospora* in the respiratory sample by culture, obtaining a sensitivity and specificity of 90.48% and 79.30%, respectively; positive and negative predictive values of 54.81% and 96.77%, and an efficiency of 81.72%. The clinical factors associated with the results were also studied using a univariate and a multivariate analysis, which showed that *Scedosporium*/*Lomentospora* positive sputum, elevated anti-*Aspergillus* serum IgG and chronic *Pseudomonas aeruginosa* infection were significantly associated with a positive result in DIA, while *Staphylococcus aureus* positive sputum showed a negative association. In conclusion, the test developed can offer a complementary, rapid, simple and sensitive method to contribute to the diagnosis of *Scedosporium*/*Lomentospora* in patients with CF.

## 1. Introduction

Species of the genera *Scedosporium* and *Lomentospora* are emerging pathogens that rank second among filamentous fungi causing chronic colonization of the airways of patients with cystic fibrosis (CF) and can lead to chronic inflammation or life-threatening invasive disease [1]. This fact makes it imperative that their diagnosis is made without delay since the prognosis worsens significantly in the absence of early detection and implementation of effective antifungal therapy.

However, currently, a definitive diagnosis of *Scedosporium*/*Lomentospora* still depends mainly on non-standardized culture methods, with low sensitivity and specificity, encompassed with data from histopathological, radiological, serological and molecular sources. These laboratory-based methods come with different limitations such as worldwide availability, turnaround times, high costs and the need for qualified staff to perform them [2], which sometimes generates a late diagnosis that contributes to treatment failure and increased morbidity and mortality.

In this sense, a new generation of point-of-care (POC) tests is gaining attention, especially with qualitative antigen-based assays, such as the ones currently available for *Aspergillus* spp. Among them, there are three main rapid tests commercially available based on Lateral Flow Devices (LFDs): “*Aspergillus* Galactomannan Lateral Flow Assay”, “*Aspergillus*-specific Lateral Flow Device Test” and “*Aspergillus* Proximity Ligation Antigen Assay”. These assays are developed to detect cell-wall antigens indicative of fungal presence, which under a given infectious symptomatology is decisive for the diagnosis of infection [3]. They can be performed on-demand, require minimal hands-on time, technical skills and equipment to operate, and provide rapid results (minutes), but unfortunately they are not available for *Scedosporium*/*Lomentospora*.

The range of options for rapid diagnostic tests includes not only the detection of circulating antigen, as in the above-mentioned methods, but also specific antibodies. Antibody-testing-based methods are particularly useful for the diagnosis of fungal conditions such as chronic and allergic pulmonary aspergillosis, and they are also used to monitor treatment response and for immunosurveillance [4].

Therefore, in this study, a fast and portable system consisting of a dot immunobinding assay (DIA) to detect IgG antibodies against *Scedosporium*/*Lomentospora* in sera from patients with CF has been designed and optimized. Moreover, univariate and multivariate statistical analyses have been employed to examine the clinical factors associated with positive and negative DIA results.

## 2. Materials and Methods

### 2.1. Human Serum Sample Collection, Characterization and Categorization

A total of 303 human serum samples corresponding to 162 CF patients were used in this study. Of them, 273 samples were obtained from the Cystic Fibrosis Center of Berlin (Berlin, Germany) and 30 from the Vall d’Hebron University Hospital (Barcelona, Spain). They were used with the approval of the local Charité—Universitätsmedizin Berlin Institutional Review Board (approval ID: EA2/211/20 and EA2/057/18) and the Ethics Committee from the University of the Basque Country (UPV/EHU) (via Material Transfer Agreement, approval ID: M30/2018/081).

Serum samples from Berlin were stored at the Robert Koch Institute (Berlin, Germany) and corresponding respiratory samples were cultivated for 14 days simultaneously on SceSel+ agar at 37 °C for the recovery of *Scedosporium*/*Lomentospora*; Guizotia abyssinica–creatinine agar (Staib agar) with penicillin and streptomycin at 26 °C for identification of *Cryptococcus* spp.; CHROMAgarTM *Candida* medium at 37 °C for differentiation of *Candida*; and Sabouraud Glucose Agar with chloramphenicol and gentamycin at 26 °C and 37 °C for the cultivation of other fungi. Viscous sputum samples were liquefied using Sputasol (SR0233A; Oxoid—Thermo Fisher Scientific, Waltham, MA, USA). Fungal species were identified using phenotypic tests and sequencing using the ITS2 region of the ribosomal rRNA genes and partial β-Tubulin (BT2, exon 2–4) gene as appropriate.

On the other hand, serum samples from Barcelona were also classified based on the results of the mycological examination of a sputum sample incubated at 37 °C for up to 15 days on Sabouraud Gentamicin Chloramphenicol agar, and simultaneously on modified Thayer Martin agar or Sabouraud Chloramphenicol agar supplemented with 0.5 g/L cycloheximide for the specific recovery of the *Scedosporium*/*Lomentospora* species. For identification at a species level, molds were identified using microscopic observation; MALDI-TOF for *Aspergillus* spp. and β-tubulin (BT2, exon 2–4) sequencing for *Scedosporium*/*Lomentospora* were conducted as appropriate. For yeasts, chromogenic media and MALDI-TOF were used, and VITEK^®^ 2 Yeast identification cards (YST) (bioMerieux, Marcy-l’Étoile, France) were used only when needed. 

### 2.2. Subject Population Determination and Research Outputs

**Figure 1:**
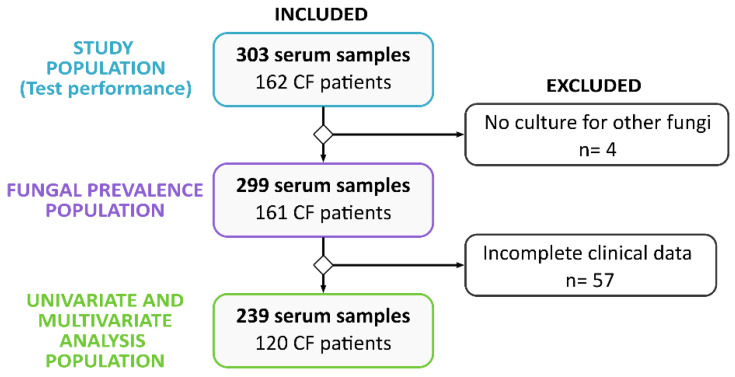
Flow diagram for study population. Dataset employed in each stage of the project (**left**): total population for test performance, for fungal prevalence calculation and for univariate/multivariate analysis. Samples excluded from evaluable dataset and reasons for exclusion (**right**).

The inclusion criteria for the initial selection of serum samples were that they derived from CF patients, and that they had data from the culture of respiratory secretions obtained on the same day of serum extraction, as well as the availability of other concomitant clinical data. From all the samples included in the study, the subject population for each specific analysis was determined based on the availability of data required (Figure 1). In this regard, an initial study population of 303 serum samples was assayed with DIA rapid tests (162 patients). 

The fungal prevalence was analyzed for 299 samples (161 patients), which were those with data available for the following microbiological cultures: *Aspergillus fumigatus* and non-*fumigatus Aspergillus* species, *Scedosporium* spp. and *Lomentospora prolificans*, and *Exophiala* spp. and *Candida* spp.

Finally, the univariate and multivariate statistical analyses were performed with the 239 samples (120 patients) that had a complete data set of the next parameters: age (years), gender (female/male), mutation of the cystic fibrosis transmembrane conductance regulator gene (CFTR mutation), body mass index (BMI) (kg/m^2^), percent-predicted mean forced expiratory volume in one second (FEV1) (%), pancreatic insufficiency (yes/no), lung transplantation (yes/no), current pulmonary exacerbation (yes/no), history of allergic bronchopulmonary aspergillosis (ABPA) (yes/no), total serum IgG (mg/L) and IgE (kU/L), *Aspergillus* serum IgG (mg/L) and IgE (kU/L) (measured by ImmunoCAP m^3^), chronic *Pseudomonas aeruginosa* colonization (yes/no), antifungal treatment (yes/no), antibiotic treatment (oral-inhaled or systemic, yes/no), sputum fungal culture result for *Aspergillus* spp., *Scedosporium*/*Lomentospora*, *Exophiala* spp. and *Candida* spp. (positive/negative), and bacterial culture result for *Staphylococcus aureus*, *Haemophilus influenzae*, *Achromobacter xylosoxidans*, *Stenotrophomonas maltophilia*, *Burkholderia multivorans* and *Mycobacterium avium* (positive/negative).

### 2.3. Dot Immunobinding Assay (DIA)

The developed rapid DIA test consisted of detection strips with printed antigenic spots to be used in a dipstick format within a miniaturized rapid immunoassay. 

#### 2.3.1. Selection of the Fungal Species and Obtaining of the Antigenic Extract

To select the fungal species for antigen extraction, the origin of the CF serum samples (Spain and Germany) was considered. The latest epidemiological study in CF patients in Germany [5] reports *S. boydii* as the most isolated *Scedosporium*/*Lomentospora* species. Regarding Spain, there are no studies conducted on CF patients discriminating between species within these genera, but in the nearby country France, *S. boydii* is also the most prevalent species [6]. Moreover, a crude antigenic extract of this fungus was previously analyzed by our research group and demonstrated to be useful in discriminating *Scedosporium*/*Lomentospora* positive CF patients by ELISA [7]. Therefore, a whole-cell protein (WCP) extract of *S. boydii* conidia and hyphae was obtained following the protocol optimized by our research group. Briefly, the fungus was grown for 24 h at 37 °C and 120 rpm in potato dextrose broth (PDB) (Condalab, Spain). Fungal growth was recovered by filtration, washed with phosphate buffered saline at pH 7.4 (PBS), resuspended in PBS supplemented with 1% (*v*/*v*) b-mercaptoethanol and 1% (*v*/*v*) ampholytes at pH 3–10 (GE Healthcare, Solingen, Germany) and disrupted by bead-beating for 20 min at 30 Hz using theMillMix20 (Domel, Železniki, Slovenia). Cell debris was discarded by centrifugation, and the resulting WCP suspension was sonicated and stored at −80 °C. 

#### 2.3.2. Printing and Functionalization of Detection Strips

**Figure 2:**
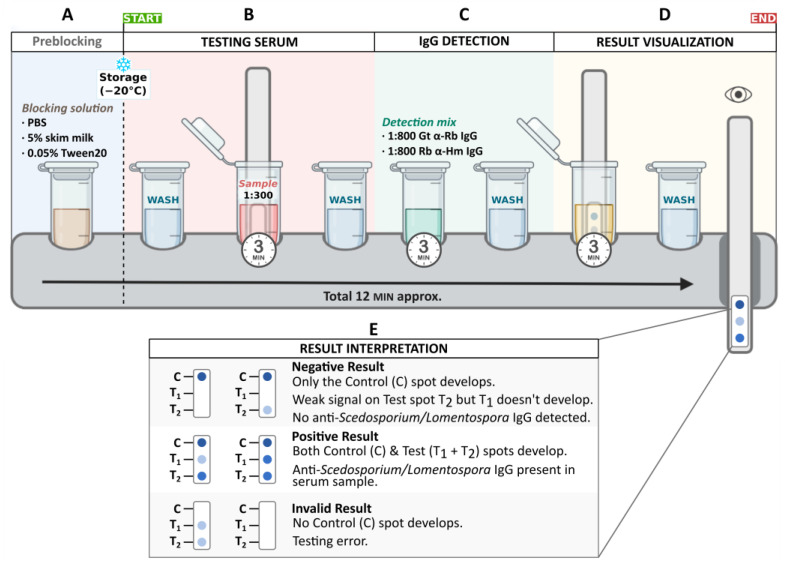
Testing procedure. Rapid immunoassay protocol starting from preblocked strips (**A**), 3 min incubation with testing serum sample (**B**), IgG detection with 3 min incubation with detection mix (**C**), soaking in TMB substrate for development of color (**D**) and visual interpretation of possible results (**E**).

WCP extract was immobilized over acetate strips using the non-contact spotting robot NanoPlotter NP2.1 (GeSiM, Radeberg, Germany) under controlled conditions of 65% relative humidity. To achieve that, the fungal extract was diluted with SOLPP imprint solution (Functionalization solution; IMG Pharma Biotech S.L., Derio, Spain) to a final concentration of 80 µg/mL and 320 µg/mL. Using nano-printing technology, both dilutions were dotted independently over the polymeric surface by printing two droplets of 30 nL per spot with a solenoid valve pipetting tip, immobilizing a total of 4.8 µg (“T1” on Figure 2E) and 19.2 µg (“T2” on Figure 2E) of protein extract on each testing spot. Moreover, 0.5 ng of purified human IgG (I4506; Sigma-Aldrich, St. Louis, MO, USA) was also dotted onto the strip to act as test control (“C” on Figure 2E).

After a desiccant drying process to ensure the correct fixation of proteins, detection strips were first washed with PBS and afterwards functionalized by soaking in blocking solution (5% [*w*/*v*] skim milk powder, 0.05% [*v*/*v*] Tween 20 in PBS) for 1 h at RT. To ensure the maintenance of functional activity, detection strips were washed again with PBS, air-dried and preserved under dry conditions at −20 °C until use.

#### 2.3.3. Three Dimensional (3D) Design of Backings and Testing Rack

Detection strips were incorporated into a 3D printed cartridge to provide robustness and facilitate their usability. The 3D design was first created with Autodesk 123D software (Autodesk, California, USA) and afterwards processed with Cura software (Ultimaker, Utrecht, The Netherlands) to be ready for printing. On the one hand, backings were 3D printed in polylactic acid (PLA) biodegradable polyester using a Prusa i3 mk3printer (Prusa Research, Prague, Czech Republic) with a 0.6 mm nozzle and 0.4 mm base layer height. On the other hand, a testing rack with hermetic cuvettes for every step of the immunoassay was printed in resin using a Photon MonoX printer (Anycubic, Shenzhen, China). These 3D printed pieces were designed and produced in collaboration with the Egokitek 3D Company (Donostia-San Sebastian, Spain).

#### 2.3.4. Immunoassay Procedure

Detection strips were used as anti-*Scedosporium*/*Lomentospora* IgG capture surfaces in a miniaturized immunoassay, following the detailed procedure shown in Figure 2. Briefly, a plastic backing containing one pre-blocked detection strip (one test) was first washed by dipping it in PBS containing 0.05% (*v*/*v*) Tween 20 (PBST), and then immersed in the serum sample diluted 1:300 in blocking solution for 3 min. After dipping and washing in PBST to avoid the nonspecific binding, the strip was soaked for 3 min in detection dolution (premixed HRP-labelled Goat Anti-Rabbit IgG [ab6721; Abcam, Cambridge, UK] 1:800 and HRP-labelled Rabbit Anti-Human IgG [ab6759; Abcam, Cambridge, UK] 1:800 in PBST). Final dipping washings preceded and incubation of 3 min in a 3,3′,5,5′ Tetramethylbenzidine (TMB) liquid substrate system followed (T0565; Sigma Aldrich). Visual naked-eye detection of a blue dot on both test lines (T1 and T2) and the control (C) means a positive result (Figure 2).

### 2.4. Data Processing, Statistical Treatment and Analysis

#### 2.4.1. Test Performance

The DIA test performance was evaluated by comparison with the result of the *Scedosporium*/*Lomentospora* detection from a culture of respiratory samples, calculating validation parameters of sensitivity (SE), specificity (SP), positive predictive value (PPV), negative predictive value (NPV), efficiency (EFF), likelihood ratio of positive and negative test (LR+ and LR−) and diagnostic odds ratio (OR). Likewise, Cohen’s Kappa chance corrected index of agreement was also calculated (K).

#### 2.4.2. Univariate Analysis

The DIA test results (positive/negative) were compared with the above-mentioned clinical parameters using univariate analyses. The distribution of the data was assessed with the Shapiro–Wilk test for normal distribution.

For metrical variables, median and ranges of positive and negative groups were calculated, and for comparison between them, *t*-test or Mann–Whitney U test was applied as appropriate. For categorical variables, frequency and percentages were used, and statistical analysis was performed using the Chi-square test, except for variables with less than 30 cases when Fisher’s exact test was used. 

Finally, two descriptors were calculated to interpret the effect size: Cohen’s d and Phi for metrical and categorical variables, respectively. SPSS Statistics software version 24 (IBM, New York, USA) was used to perform the above-mentioned analyses. A *p*-value of < 0.05 in a two-sided test was accepted as indicator of statistical significance.

#### 2.4.3. Multivariate Analysis: Hierarchical LR Regression Model

To identify factors independently associated with positive DIA results, a hierarchical multinominal logistic regression model was built employing the *mnrfit* function in MATLAB (MathWorks, Massachusetts, USA). Except for gender and CFTR mutation, all the clinical parameters mentioned in Section 2.2 of this manuscript were adjusted and considered within the regression model, and chosen for a final model using stepwise backward variable selection, removing the least significant variable at each iteration for the adjustment. Age data was segmented in ranges of years (≤9/10–19/20–29/30–39/40–49/≥50) before inclusion in the model. OR and low and high confidence limits (LCL, HCL) were calculated. A *p*-value of <0.05 was considered statistically significant.

## 3. Results

### 3.1. DIA Results: Detection of Anti-Scedosporium/Lomentospora IgG in Human CF Sera

**Figure 3:**
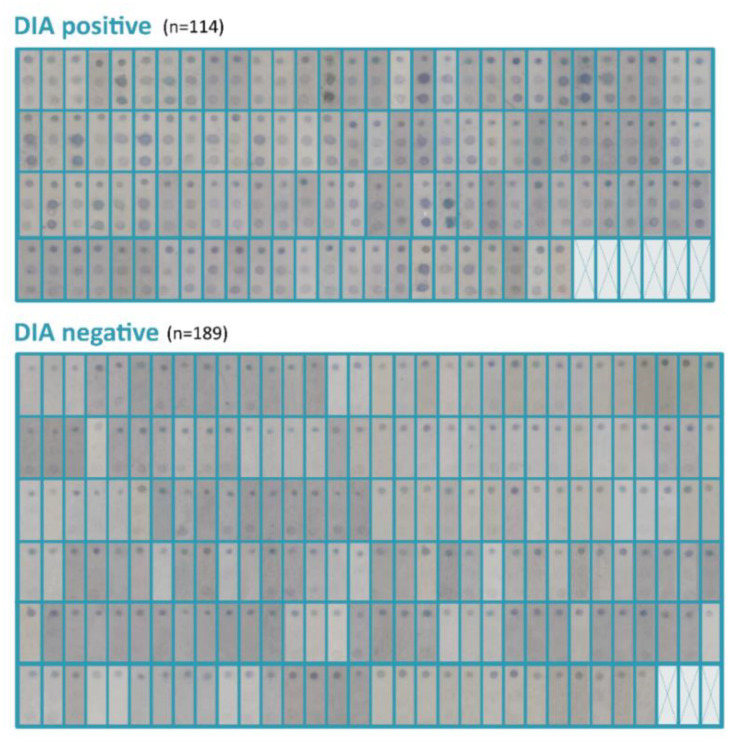
Imaging of DIA testing results. Graphical evidence of DIA tests carried out with 303 CF serum samples; 114 of them yielded a positive result for anti-*Scedosporium*/*Lomentospora* serum IgG, while 189 were negative for the rapid test.

A total of 303 serum samples were assayed with the DIA rapid test (Figure 3) and compared with culture results to determine the test performance. The results reported an SE and SP of 86.36% and 75.95%, respectively; PPV and NPV were 50% and 95.24%, and EFF was 78.22%. LR+ and LR− tests were 3.59 and 5.57, respectively, and diagnostic OR was 20. Finally, the Kappa index calculated was 0.49, which indicates moderate agreement. Test evaluation results are summarized in Table 1.

The detailed results showed that 57 of the 66 *Scedosporium*/*Lomentospora* culture-positive samples were positive with DIA, but also 57 of 237 culture-negative samples. In the cases in which the reference standard result of fungal culture was different from that obtained with the DIA test, the clinical history concerning those controversial samples was studied to look for evidence of previous colonization and possible antibody remnants. In this sense, 10 out of the 57 *Scedosporium*/*Lomentospora*-culture-negative samples that were positive for DIA showed positive repetitive isolation of *Scedosporium*/*Lomentospora* from sputum samples previous to that sporadic negative sample. On the other hand, 3 of the 9 *Scedosporium*/*Lomentospora*-culture-positive samples that were negative for DIA corresponded to sporadic isolations just in the day of culture, not repeated in either previous or in subsequent sample analyses of the same patient. Fungal culture data obtained from the clinical history of the controversial samples is detailed in Table 2. 

In view of these results, these 13 samples were removed from the analysis and validation parameters were recalculated. As a result, SE and SP values improved to 90.48% and 79.30%, respectively. PPV and NPV were also raised to 54.81% and 96.77%, and the efficiency of the test was enhanced to 81.72% (Table 2). In this study, transplant patients have not been excluded because, although they could have an aberrant immune response, no interference in the results related to them was detected (data not shown).

### 3.2. Fungal Frequencies on Corresponding Sputum Samples

In a ranking of frequencies from the highest to the lowest, *Candida* yeasts were isolated from 200 of the 299 samples with data available for all microbiological cultures (66.9%), the most prevalent being *C. albicans* (*n* = 124, 62%), followed by *C. dubliniensis* (*n* = 69, 34.5%), *C. glabrata* (*n* = 40, 20%) and *C. parapsilosis* (*n* = 10, 5%). 

Regarding molds, *Aspergillus* spp. was recovered from 94 samples (31.44%), with *A. fumigatus* being by far the most frequent one (*n* = 89, 94.68%). Meanwhile, other recognized species such as *A. flavus* or *A. terreus* exhibited very low frequencies (*n* = 3, 3.5% for both species). A total of 69 samples (23.08%) were positive for *Scedosporium*/*Lomentospora*. Within this group, species of the *Scedosporium apiospermum* complex were the most frequently isolated (*n* = 62, 89.86%) and *S. apiospermum sensu stricto* the most prevalent (*n* = 40, 57.97%). Eight samples were positive for *L. prolificans* (11.59%) and four for *Pseudallescheria ellipsoidea* (5.8%). Finally, the black yeast *Exophiala* was detected in 36 samples (12.04%), with *Exophiala dermatitidis* being almost exclusively recovered (*n* = 34, 94.4%), as *Exophiala phaeomuriformi* was isolated from only two samples (5.56%).

**Figure 4:**
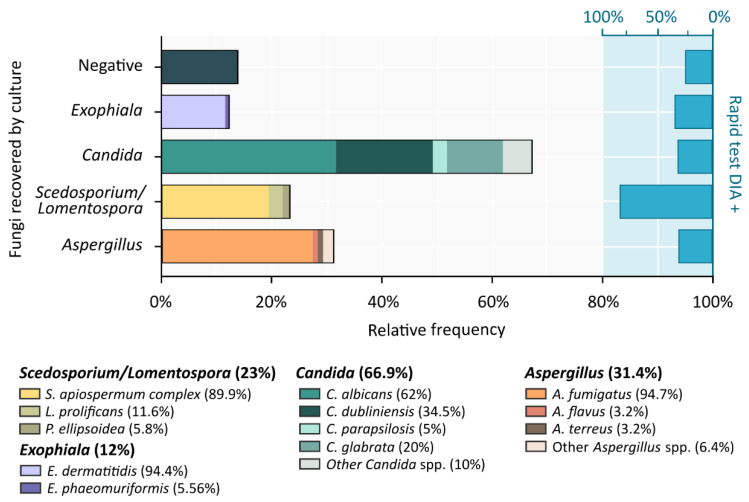
Frequency of isolation of fungal species from respiratory samples and anti-*Scedosporium*/*Lomentospora* IgG prevalence. Left y-axis and lower x-axis: relative frequencies of fungi recovered from culture of 299 sputum samples corresponding to CF patients’ tested sera. Right y-axis and upper x-axis: prevalence rates for each fungal group of anti-*Scedosporium*/*Lomentospora* IgGs present in serum assessed with DIA rapid test.

The existence of polyfungal samples is noteworthy, from which more than one fungal species was isolated at a time. In this sense, co-incidence of *Candida* and *Scedosporium*/*Lomentospora* was observed in 22 samples (7.36%); *Aspergillus* and *Scedosporium*/*Lomentospora* in 15 samples (4.35%); meanwhile, *Exophiala* and *Scedosporium*/*Lomentospora* were not recovered together in any sample. On the other hand, no fungal microorganisms were isolated in 42 samples (14.05%). The fungal prevalence in sputum samples are summarized in Figure 4.

Looking at the DIA+ ratios within each fungal group, the highest rates were indeed found in samples positive for the *Scedosporium*/*Lomentospora* culture (82.61%, *n* = 57), while DIA+ test frequencies observed for the other fungal groups were 33.3% (*n* = 12) for *Exophiala*, 30.5% (*n* = 61) for *Candida*, and 29.8% (*n* = 28) for *Aspergillus*.

### 3.3. Cohort Characteristics for Statistical Analysis

Demographic, clinical and microbiological characteristics of the cohort used for statistical analysis are summarized in Table 3. Of all the samples, 140 came from females and 99 from males. The median age of the patients was 26 years (range 7–70). Delta F508 was the most frequent CFTR mutation with a prevalence of 86.19% (homozygous mutation 63.6%, heterozygous 36.4%, other genotypes 13.80%). The median predicted FEV1% was 49 (range 16.3–123.9) and median BMI was 19.4 (range 12.4–33.7). Most of the subjects had pancreatic insufficiency (*n* = 229, 95.8%) and showed current pulmonary exacerbation (*n* = 177, 74%). A small number were lung transplanted on dates close to sampling (*n* = 8, 3.3%). ABPA history was described for 37.2% (*n* = 89). The median total serum IgG and IgE were 14.5 kU/L (range 0–281) and 48.1 kU/L (range 2–2955), respectively. The median *Aspergillus* IgG and IgE were 69 mg/L (range 2.7–200) and 0.3 kU/L (range 0.1–173) each, respectively. A total of 25.5% of patients were under antifungal treatment (*n* = 61) and 46.4% were being treated with antibiotic therapy (oral or inhaled 4.2%, systemic 42.4%). Table 3 also gives an overview of bacterial and fungal isolates in the total sera cohort, in the group of samples with a negative result for the DIA test and in those with a positive result. Fungal prevalence rates are better described in the previous section within the exhaustive analysis of fungal species isolates carried out with a higher number of samples. Regarding bacterial cultures, the highest prevalence was observed for chronic *P. aeruginosa*, with 66.5% (*n* = 159) for the total cohort, followed by 29.7% (*n* = 71) of *S. aureus* positive sputum. Other bacterial isolates were less prevalent in sputum samples: *H. influenzae* 2.9% (*n* = 7), *A. xylosoxidans* 7.5% (*n* = 18), *S. maltophilia* 3.8% (*n* = 9), *B. multivorans* 3.3% (*n* = 8) and *M. avium* 2% (*n* = 5). 

### 3.4. Association Factors for Scedosporium/Lomentospora-IgG DIA Positive Test

**Figure 5:**
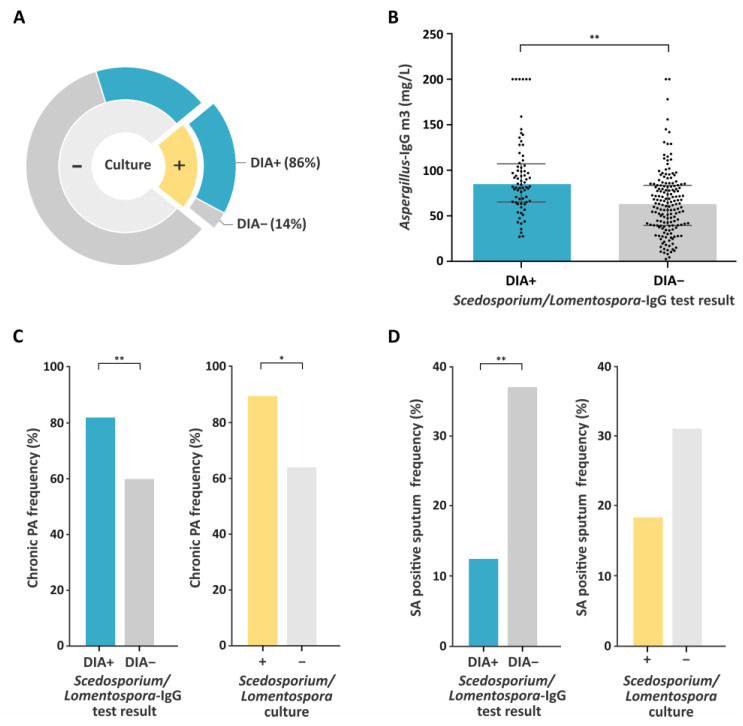
Association factors for positive *Scedosporium*/*Lomentospora*-IgG rapid test (DIA). Variables identified in univariate analysis and adjusted multivariate analysis as significantly associated with positive *Scedosporium*/*Lomentospora*-IgG: Frequency of DIA+ results in *Scedosporium*/*Lomentospora* positive cultures (**A**), *Aspergillus* IgG levels (**B**), chronic *P. aeruginosa* frequency (**C**) and *S. aureus* positive sputum frequency (**D**). Blue and yellow relate to DIA test result and *Scedosporium*/*Lomentospora* culture result, respectively. Dark and light grey relate to DIA negative and *Scedosporium/Lomentospora* negative culture, respectively. PA: *P. aeruginosa*, SA: *S. aureus*. * *p* < 0.05; ** *p* < 0.001.

Univariate analyses revealed that *Scedosporium*/*Lomentospora* positive sputum incidence was higher within the DIA+ group (Table 3, Figure 5A). This is an expected result since the developed test was designed to detect IgG against these fungi and, as a consequence, this association showed the highest significance (*p* = 7.0588 × 10 ^−8^). 

In addition, *Aspergillus* IgG levels were significantly higher in DIA+ (84.9 mg/L, range 27.1–200, *p* = 2.1157 × 10 ^−7^) (Figure 5B), as was the frequency of chronic *P. aeruginosa* (*n* = 59, 81.9%, *p* = 0.000912) (Figure 5C). Conversely, the frequency of *S. aureus* positive sputum was significantly higher in the DIA− sera cohort (*n* = 62, 37.1%, *p* = 0.000132) (Figure 5D). Nevertheless, these *P. aeruginosa* and *S. aureus* data do not seem to be directly correlated with the DIA IgG rapid test result, but with the presence (detected by culture) of *Scedosporium*/*Lomentospora* fungi themselves (Figure 5C,D). In fact, chronic *P. aeruginosa* incidence was significantly higher in patients with positive cultures for *Scedosporium*/*Lomentospora*. On the other hand, *S. aureus* was recovered less from sputum samples positive for *Scedosporium*/*Lomentospora* culture, although differences in this case were not significant. Finally, systemic antibiotic therapy was significantly more frequent in the DIA− group (*n* = 78, 46.7%, *p* = 0.034). 

Demographic, clinical and microbiological association factors correlating with a *Scedosporium*/*Lomentospora*-IgG positive test calculated using a hierarchical multinominal logistic regression model are shown in Table 4. The adjustment of variables is specified according to the criteria detailed in Section 2. The following variables identified from the univariate analysis were confirmed using adjusted multivariate analysis as being statistically significantly associated with positive *Scedosporium*/*Lomentospora*-IgG: *Aspergillus* serum IgG (OR 18.6229, 95% CI: 8.3412 ± 41.5786, *p* = 5.52 × 10 ^−7^), *Scedosporium*/*Lomentospora* positive sputum (OR 3.1569, 95% CI: 2.3040 ± 4.3255, *p* = 9.45 × 10 ^−7^) and chronic *P. aeruginosa* (OR 27.5843, 95% CI: 8.8439 ± 86.0362, *p* = 0.046). However, *A. xylosoxidans* positive sputum was also detected as a significant association factor for positive *Scedosporium*/*Lomentospora*-IgG (OR 3.9814, 95% CI: 1.6160 ± 9.8092, *p* = 0.017) in the logistic regression model built, while no significant differences were observed for this variable between the DIA− and DIA+ groups in the previous descriptive study. Correlating with univariate analysis, *S. aureus* positive sputum was also identified by the model as negatively associated (OR < 1) with a positive *Scedosporium*/*Lomentospora*-IgG test (OR 0.0693, 95% CI: 0.0249 ± 0.1930, *p* = 0.006).

## 4. Discussion

In the present work, a rapid, sensitive and portable serological test, which allows the detection of anti-*Scedosporium*/*Lomentospora* IgG antibodies in serum from patients with CF in a few minutes is presented. This system meets most of the ASSURED criteria (Affordable, Sensitive, Specific, User-friendly, Rapid and Robust, Equipment fee, Deliverable to end users) established by the World Health Organization (WHO) as a benchmark for the successful development of diagnostics in all income and resource settings [8].

To design this DIA system, anti-*Scedosporium*/*Lomentospora* IgG detection strips were developed, employing *S. boydii* WCP extract as an antigen, to obtain a visual reading of the results in only 12 min. This immunoassay shows a promising performance when compared to results of the “gold standard” method used to detect these fungi in CF patients, the culture of the respiratory secretions (SE = 86.36%, SP = 75.95%, PPV = 50%, NPV = 95.24%, EFF = 78.22%). These parameters represent a breakthrough in *Scedosporium*/*Lomentospora* serodiagnosis in comparison with those obtained with the ELISA previously presented [7], since it allows the obtaining of results in a few minutes, maintaining good validation parameters. 

The weaker PPV results obtained might be explained due to a misclassification of sera. In fact, considering the lack of standardization and sensitivity of the mycological examination of sputum samples [2,9], some sera might have been classified as negative because no *Scedosporium*/*Lomentospora* was recovered from their sputum sample, even though they may have been infected or colonized. On the other hand, sporadic isolation does not imply infection, as it can be the result of transient colonization that has not triggered an adaptive immune response. In this sense, it is vital to analyze the history of the patients and check for repeated isolations to classify the serum. In fact, 13 samples were detected as misclassified, and after their removal, validation parameters improved to 90.48% for SE, 79.30% for SP, 54.81% for PPV and 96.77% for NPV. It should be also mentioned that there was no previous clinical record available for the 20 samples with contradictory results for DIA and culture, so it cannot be discounted that there were more misclassifications. In the future, it would be interesting to perform a larger-scale multicenter study, which would allow an large enough number of samples, but with more restrictive and standardized inclusion criteria, following the latest guidelines proposed for fungal diagnosis [10,11,12]. Moreover, criteria to differentiate colonization and infection might also be included with group positive sera [12,13] and, in this way, decipher whether the DIA test is able to discriminate between them.

Regarding the fungal prevalence in the sputum samples associated with the sera used, *C. albicans* was the most frequently isolated yeast (41.5%) and *A. fumigatus* the most prevalent mold (29.86%), followed by the *S. apiospermum* complex species (20.7%) and *E. dermatitis* (11.37%). These frequencies are in line with the ranking described in different epidemiological studies, such as the data reported in the MucoFong International Project “MFIP” [14]. Within *Scedosporium*/*Lomentospora*, *L. prolificans* was isolated from a very small number of samples (2.7%) compared with the prevalence observed in a Spanish cohort that reached 8% [7]. Considering that the majority of the samples were obtained from a German CF center, and few of them from Spain, the low prevalence observed for *L. prolificans* could be explained, to some extent, by the reported geographical restriction of this species, mainly to Spain and Australia [15].

In addition to the test performance, the present work describes the association factors for *Scedosporium*/*Lomentospora* seropositivity in a DIA test. Univariate analysis of baseline clinical characteristics and adjusted multivariate analysis revealed that, as expected, patients with *Scedosporium*/*Lomentospora* positive sputum culture showed a statistically significant higher probability of giving a positive result in the DIA test. In addition, chronic *P. aeruginosa* and increased levels of *Aspergillus* IgG showed the same correlation. On the other hand, *S. aureus* positive sputum was negatively correlated with a positive DIA test result. Nevertheless, analyzing these variables in comparison with *Scedosporium*/*Lomentospora* culture results, it was concluded that *P. aeruginosa* and *S. aureus* data were also positively and negatively correlated, respectively, with the presence in the sputum culture of *Scedosporium*/*Lomentospora*.

The available bibliography reports similar results for these association factors in the sera collection used for testing an in-house ELISA test to detect *Scedosporium*/*Lomentospora* [16]. In fact, the high coexistence of *Pseudomonas* and *Scedosporium*/*Lomentospora* has also been supported by a prospective German multicenter trial [5], a study of a cohort of CF patients from Spain [17], a retrospective cohort study of CF subjects from the United States [18] and a Dutch cross-sectional study [19]. On the other hand, in vitro studies showed that *P. aeruginosa* inhibits the growth of *Scedosporium*/*Lomentospora* [20,21,22] but, interestingly, Homa and colleagues deciphered that while in direct physical contact, *P. aeruginosa* inhibited the growth of *Scedosporium* spp., and when cultured in non-direct contact conditions, *Scedosporium* growth was stimulated by the production of bacterial signal molecules [23]. Regarding the higher frequency of *S. aureus* in *Scedosporium*/*Lomentospora* negative samples, other authors have also shown similar results in different in vivo studies [16,17,18]. Moreover, *S. aureus* produces some metabolites that display an antifungal effect on *S. apiospermum* and *S. boydii* [23]. Likewise, peptidorhamnomannans from the surface of *Scedosporium* spp. inhibit the growth and biofilm formation of *S. aureus* [24], and some secondary metabolites, like polyketide boydone A, secreted by *S. boydii,* have been reported to exhibit anti-*S. aureus* activity [25]. Thus, it may also contribute to the lower coexistence rates of these two pathogens.

**Figure 6:**
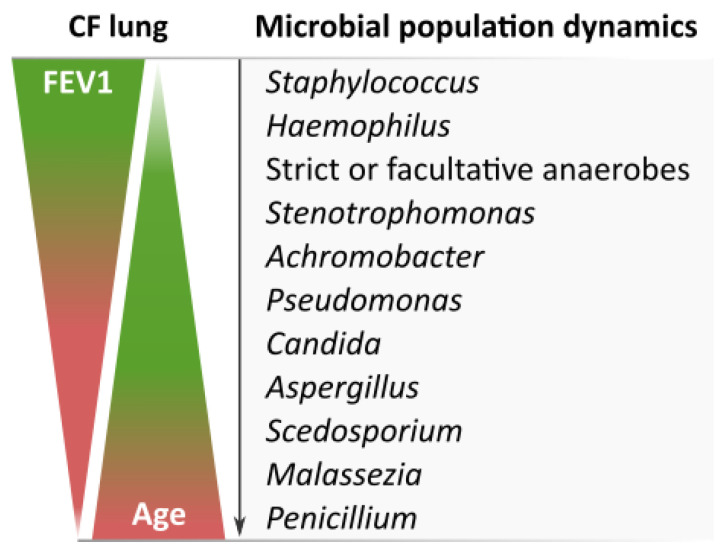
Evolution of microbial communities in the CF lung (according to [26]). The most relevant bacteria and fungi are named in order of settlement.

These results are in line with the evolution of the microbial and fungal communities during the progression of CF (Figure 6) [26], with *S. aureus* being more prevalent during the early onset of the disease, and *Pseudomonas* in young adults when CF has progressed and fungal species such as *Scedosporium*/*Lomentospora* start to appear [27]. In fact, colonization of the airways by *Scedosporium*/*Lomentospora* occurs more frequently in CF adolescents and adults than in children, and often later than colonization by *Aspergillus* spp. [28].

To finish with factors related to DIA positive results, the association between elevated *Aspergillus* IgG levels and *Scedosporium*/*Lomentospora* detection from culture was also previously observed and explained by the common occurrence of mixed colonization due to the sharing of ecological niches and the arising immune cross-reactions [16]. In relation to this, in a previous study, a total protein extract of *A. fumigatus* was not able to discriminate *Aspergillus* positive patients from those with *Scedosporium*/*Lomentospora*, whereas *S. boydii* WCP extract succeeded in discriminating *Scedosporium*/*Lomentospora* positive patients from those with *Aspergillus* [7]. Therefore, it might be inferred that the association between elevated *Aspergillus* IgG levels and *Scedosporium*/*Lomentospora* presence is related to a cross-reactivity with the *A. fumigatus* antigenic extract used to determine anti-*Aspergillus* IgG levels, rather than with the *S. boydii* WCP extract used in the DIA developed in this study. 

Many clinical and epidemiological studies also point out the relevance of other bacteria from the climax population, such as *A. xylosoxidans* and *H. influenzae* [5,17], although their results are contradictory. This study detected *A. xylosoxidans* as an association factor for *Scedosporium* seropositivity using the adjusted multivariate model, but not from the univariate analysis. Regarding *H. influenza*, although a higher frequency in the DIA− population was observed, the sample size was very limited and differences were not significant.

The prolonged use of antibiotics has also been extensively associated with a higher risk of fungal colonization [14,15,29] and, specifically, inhaled antibiotic treatment with *Scedosporium*/*Lomentospora* isolation in CF patients [18,19]. The data presented here also showed that the frequency of oral or inhaled antibiotics was higher, but not significant, in the *Scedosporium*/*Lomentospora* DIA positive group. In the same way, a correlation has also been described between ABPA and *Scedosporium* seropositivity [5,16,30], but it was not detected by our model, probably due to the limited number and older age of patients compared to that associated with *Scedosporium*/*Lomentospora* colonization [5].

Finally, this study has a limitation concerning the method used to compare the DIA results and carry out the test performance, because although the culture of respiratory samples has been commonly used [2,31], it is not a good parameter for being used in isolation as the “gold standard” for the evaluation of the performance of a serological test. In fact, each method is used to evaluate different things. At best, culture detects presence but is not necessarily indicative of infection or tissue invasion, whereas the detection of IgG (DIA test) is related to the host immune response, indicating more than a mere fungal presence or colonization without tissue repercussions. In the case of *Scedosporium*/*Lomentospora* in a non-immunocompromised patient, such as those suffering from CF, the functional impact (deterioration of respiratory function) generally develops slowly, in a chronic and latent form, and by the time they are detected, it is usually too late. This DIA test could help to anticipate the onset of these functional repercussions, similar to the detection of anti-*A. fumigatus* IgGs in chronic aspergillosis [32,33]. 

In conclusion, the rapid serological test developed in this work is a useful tool that allows the detection and monitoring of the IgG-mediated humoral response of CF patients against *Scedosporium*/*Lomentospora* with good SE and SP. Due to the characteristics of the test, the low hands-on time and simplicity of the protocol, the DIA test is a diagnostic tool that could even be part of primary care equipment, improving the surveillance of CF patients. 

## Figures and Tables

**Table 1:** Test performance. Contingency tables comparing the results of the DIA rapid test for anti-*Scedosporium*/*Lomentospora* specific IgG and result of sputum culture for *Scedosporium*/*Lomentospora* of 303 CF samples. Test validation parameters of performance, index and estimate.

Culture Result	DIA Rapid Test Result		Total	Test Performance	
	Positive	Negative		Index	Estimate
Positive	57 (18.81%)	9 (2.97%)	66 (21.78%)	SE/SP (%)	86.36/75.95
Negative	57 (18.81%)	180 (59.40%)	237 (78.22%)	PPV */NPV *(%)	50/95.24
Total	114 (37.62%)	189 (62.38%)	303 (100%)	EFF (%)	78.22
				LR+/LR−	3.59/5.57
				OR	20
				K	0.49

SE: sensitivity, SP: specificity, PPV: predictive value of positive test, NPV: predictive value of negative test, EFF: efficiency of correct classification rate, LR+: likelihood or risk ratio of positive test, LR−: likelihood or risk ratio of negative test, OR: overall odds ratio, K: Cohen’s Kappa index. (*) Theoretical values considering a prevalence of *Scedosporium/Lomentospora* of 21.78% observed in this sera cohort.

**Table 2:** *Scedosporium*/*Lomentospora* history of controversial samples (non-coincident result between culture and DIA test) and new test performance removing justified samples.

Culture Positive/DIA Negative	N	Test Performance	
Total	9	**Index**	**Estimate**
Single-sporadic isolation	3	SE/SP (%)	90.48/79.30
Positive history	4	PPV */NPV * (%)	54.81/96.77
No record available	2	EFF (%)	81.72
**Culture negative/DIA positive**	**N**	LR+/LR−	4.37/8.32
Total	57	OR	36.38
Positive history	10	K	0.56
Negative history	29		
*Candida*	12		
*Aspergillus* and *Candida*	6		
*Aspergillus*	2		
*Aspergillus*, *Exophiala* and *Candida*	3		
*Exophiala* and *Candida*	1		
*Exophiala*	1		
No fungi recovered	4		
No record available	18		

All cultures and histories correspond to *Scedosporium*/*Lomentospora* spp. unless otherwise specified. Samples justified for removal are highlighted in grey. SE: sensitivity, SP: specificity, PPV: predictive value of positive test, NPV: predictive value of negative test, EFF: efficiency of correct classification rate. (*) Theoretical values considering a prevalence of *Scedosporium*/*Lomentospora* of 21.72% observed in this sera cohort.

**Table 3:** Baseline clinical characteristics of patients’ samples. Total samples, negative (DIA-) and positive (DIA+) samples for the rapid test.

Variable	Total	DIA−	DIA+	*p*-Value	Effect Size
Number of tested serum samples, *n* (%)	239	167 (69.87%)	72 (30.13%)		
Age, years, median (range)	26 (7–70)	26 (7–53)	29 (9–70)	0.329	0.144 a
Gender, female sex, *n* (%)	140 (58.6%)	97 (58.1%)	43 (59.7%)	0.813	0.015 b
CFTR dF508 homozygous, *n* (%)	152 (63.6%)	102 (61.1%)	50 (69.4%)	0.217	0.080 b
BMI, kg/m^2^, median (range)	19.4 (12.4–33.7)	19.3 (12.5–33.7)	19.7 (12.4–26.9)	0.870	0.202 a
Percent-predicted FEV1, median (range)	49 (16.3–123.9)	51.4 (16.3–123.9)	46.8 (17–101)	0.352	−0.197 a
Pancreatic insufficiency, *n* (%)	229 (95.8%)	159 (95.2%)	70 (97.2%)	0.476	0.046 b
Lung transplantation, *n* (%)	8 (3.3%)	5 (3%)	3 (4.2%)	0.700	0.030 b
Pulmonary exacerbation, *n* (%)	177 (74%)	125 (74.9%)	52 (72.2%)	0.671	−0.028 b
ABPA history, *n* (%)	89 (37.2%)	59 (35.3%)	30 (41.7%)	0.352	0.060 b
Total serum IgG, kU/L, median (range)	14.5 (0–281)	14.5 (2–281)	14.5 (0–48)	0.211	−0.053 a
*Aspergillus* serum IgG, mg/L, median (range)	69 (2.7–200)	63.1 (2.7–200)	84.9 (27.1–200)	2.1157 × 10 ^−7^ **	0.780 a
Total serum IgE, kU/L, median (range)	48.1 (2–2955)	44.1 (2–2658)	59.75 (2–2955)	0.382	0.003 a
*Aspergillus* serum IgE, kU/L, median (range)	0.3 (0.1–173)	0.1 (0.1–173)	0.7 (0.1–31)	0.120	−0.140 a
Chronic *Pseudomonas aeruginosa*, *n* (%)	159 (66.5%)	100 (59.9%)	59 (81.9%)	0.000912 **	0.215 b
Antifungal treatment, *n* (%)	61 (25.5%)	39 (23.4%)	22 (30.6%)	0.241	0.076 b
Oral/Inhaled antibiotic treatment, *n* (%)	10 (4.2%)	4 (2.4%)	6 (8.3%)	0.070	0.136 b
Systemic antibiotic treatment, *n* (%)	101 (42.2%)	78 (46.7%)	23 (31.9%)	0.034 *	−0.137 b
*Aspergillus* spp. positive sputum, *n* (%)	86 (36%)	63 (37.7%)	23 (31.9%)	0.393	−0.055 b
*Scedosporium*/*Lomentospora* positive sputum, *n* (%)	27 (11.3%)	6 (3.6%)	21 (29.2%)	7.0588 × 10 ^−8^ **	0.371 b
*Exophiala* spp. positive sputum, *n* (%)	33 (13.8%)	22 (13.2%)	11 (15.3%)	0.665	0.028 b
*Candida* spp. positive sputum, *n* (%)	185 (77.4%)	129 (77.2%)	56 (77.8%)	0.928	0.006 b
*Staphylococcus aureus* positive sputum, *n* (%)	71 (29.7%)	62 (37.1%)	9 (12.5%)	0.000132 **	−0.247 b
*Haemophilus influenzae* positive sputum, *n* (%)	7 (2.9%)	6 (3.6%)	1 (1.4%)	0.678	−0.060 b
*Achromobacter xylosoxidans* positive sputum, *n* (%)	18 (7.5%)	10 (6%)	8 (11.1%)	0.186	0.089 b
*Stenotrophomonas maltophilia* positive sputum, *n* (%)	9 (3.8%)	9 (5.4%)	0	0.061	−0.130 b
*Burkholderia multivorans* positive sputum, *n* (%)	8 (3.3%)	3 (1.8%)	5 (6.9%)	0.056	0.131 b
*Mycobacterium avium* positive sputum, *n* (%)	5 (2%)	5 (3%)	0	0.326	−0.096 b

* *p* < 0.05; ** *p* < 0.001; a: Cohen’s d; b: Phi.

**Table 4:** Adjusted odds ratios of association factors for *Scedosporium*/*Lomentospora*-IgG DIA positive test.

Variable	OR	LCL	HCL	*p*-Value
*Aspergillus* serum IgG m3	18.6229	8.3412	41.5786	5.52 × 10 ^−7^ **
*Scedosporium*/*Lomentospora* positive sputum	3.1569	2.3040	4.3255	9.45 × 10 ^−7^ **
*Staphylococcus aureus* positive sputum	0.0693	0.0249	0.1930	0.006 *
*Achromobacter xylosoxidans* positive sputum	3.9814	1.6160	9.8092	0.017 *
Chronic *Pseudomonas aeruginosa*	27.5843	8.8439	86.0362	0.046 *
*Burkholderia multivorans* positive sputum	0.1528	0.0419	0.5569	0.054
*Haemophilus influenzae* positive sputum	0.1905	0.0383	0.9472	0.142
Total serum IgE	0.3737	0.1468	0.9515	0.224
*Stenotrophomonas maltophilia* positive sputum	0.1398	0.0367	0.5333	0.257
Oral/Inhaled antibiotic treatment	0.3786	0.1452	0.9875	0.261
*Aspergillus* spp. positive sputum	0.9728	0.9619	0.9840	0.268
Pancreatic insufficiency	14.7062	1.3674	158.1603	0.270
Lung transplantation	13.6497	1.2718	146.5003	0.326
Antimycotic treatment	0.3916	0.0799	1.9194	0.473
Percent-predicted FEV1	0.3616	0.0738	1.7721	0.484
ABPA history	0.3176	0.0618	1.6327	0.502
Pulmonary exacerbation	0.4040	0.1517	1.0762	0.530
*Candida* spp. positive sputum	0.0456	0.0135	0.1540	0.589
*Exophiala* spp. positive sputum	0.0427	0.0118	0.1551	0.653
*Aspergillus* serum IgE m3	1.0004	0.9990	1.0018	0.728
BMI	1.2876	0.5140	3.2255	0.736
Total serum IgG	1.0101	0.9539	1.0695	0.772
Systemic antibiotic treatment	1.6377	0.5860	4.5771	0.894
Age	16.7536	0.3127	897.6987	0.915
*Mycobacterium avium* positive sputum	0.3798	0.0620	2.3278	1.000

* *p* < 0.05; ** *p* < 0.001; OR: odds ratio (exponential value of β); LCL: low confidence limit; HCL: high confidence limit (LCL and HCL are exponential values of 95% confidence interval for β).

## Data Availability

The data presented in this study are included in the article; further inquiries can be directed to the corresponding author.
